# Editorial: Bridging the gap: an interdisciplinary perspective on ketamine in psychiatric disorders – volume II

**DOI:** 10.3389/fpsyt.2024.1471609

**Published:** 2024-08-30

**Authors:** Glenn Hartelius, Sherry-Anne Muscat, Lucie Bartova

**Affiliations:** ^1^ Attention Strategies Institute, Berkeley, CA, United States; ^2^ Department of Mindfulness-Based Transpersonal Counseling, California Institute for Human Science, Encinitas, CA, United States; ^3^ Department of Psychology, Naropa University, Boulder, CO, United States; ^4^ Youth Forensic Psychiatry, Alberta Hospital, Edmonton, AB, Canada; ^5^ Department of Psychiatry and Psychotherapy, Medical University of Vienna, Vienna, Austria

**Keywords:** ketamine for depression, ketamine-assisted psychotherapy, intranasal esketamine, treatment-resistant depression (TRD), ketamine treatment protocols, antianhedonic effect, non-pharmacological adjunctives, ketamine placebo effect

As with our previous volume (1), this collection of papers presents emerging research and perspectives on understudied topics related to the use of ketamine for psychiatric diseases. The first three papers focus on ketamine-assisted psychotherapy, the last of these and the next two consider the impact of ketamine as an effective treatment in psychiatric indications beyond major depressive disorder (MDD). The final four articles provide practical recommendations for on-label use of the nasal administration of the esketamine enantiomer in clinical routines, consideration of non-pharmacological adjunctives, potentials for use with prison populations, and the substantial role of placebo response in ketamine study participants.

Ketamine is most often applied within a treatment framework carried over from conventional antidepressants, a model in which a drug that impacts symptoms of depression is administered at regular intervals. Yet in several ways ketamine is not a conventional antidepressant: its effects on symptoms of depression are rapid and robust; side effects are typically mild, transient, and limited to the administration period; and depending on route of administration and subanesthetic dose, ketamine typically produces alterations of conscious experience from mild mood elevation to non-serotonergic psychedelic experiences. Besides the approved intranasal formulation of esketamine for treatment resistant depression (TRD) and psychiatric emergency situations in MDD, there is growing evidence of promising effects beyond this diagnosis. This is attributable to its multifaceted pharmacological profile including glutamatergic action and secondary monoaminergic, GABAergic, opioid- and immunomodulatory effects (2). When treatment protocols are designed to integrate and harness the full range of ketamine’s intrinsic properties, ketamine therapies can realize their full potentials (3), possibly leading to improved outcomes in broader patient populations.

Our first paper, “The Montreal model: An integrative biomedical-psychedelic approach to ketamine for severe treatment-resistant depression,” takes this path toward optimizing the use of ketamine’s unique potentials. Garel et al. have developed clinical approaches that work to bridge biomedical and psychedelic models of care by combining clinician-guided ketamine sessions and nonpharmacological adjunctives such as music, blindfolds, and calm, de-medicalized environments with low intravenous doses (0.5 – 1.0 mg/kg) typical of biomedical protocols. While this paper does not report on patient outcomes, it presents a detailed and well-considered effort to bridging gaps between conventional and alternative treatment approaches.

A different pairing of psychedelic and biomedical approaches is advanced in “Ketamine-assisted psychotherapy, psychedelic methodologies, and the impregnable value of the subjective—A new and evolving approach.” As counterpoint to decades of political suppression of psychedelics in the United States, Wolfson and Vaid argue that the state-altering experiences produced by ketamine are both unique and intrinsic to its therapeutic effects: by amplifying subjective consciousness in the context of symptom relief, it provides opportunities to deeply reshape self-experience. In the protocol outlined here, emphasis on patient mindset and environmental setting provides the container for psychodynamic transformation. This work is supported by clinician guidance both during and between psychedelic ketamine sessions, and enhanced by the psychological and biological effects of the drug. The approach and its therapeutic impact are illustrated through multiple clinical case examples of clients presenting with severe disorders relating to trauma and loss.

An additional case example of ketamine treatment combined with psychotherapy is offered in “Clinical case report: Considerable improvement of severe and difficult-to-treat obsessive-compulsive disorder with comorbid depression under treatment with esketamine and concomitant psychotherapy.” Kaltenboeck et al. report on sustained remission achieved in a 28-year-old male after 10 esketamine treatments with psychotherapy and mindfulness interventions conducted both during and between drug sessions. This case offers further evidence that challenging cases beyond TRD may also be responsive to ketamine, as well as illustrating how adjunctive psychosocial interventions may augment and aid in sustaining relief gained from ketamine treatment.

Ketamine may also represent an effective treatment option for additional psychiatric diagnoses. In “Short-term ketamine use in bipolar depression: A review of the evidence for short-term treatment management,” Wilkowska and Cubala review and discuss studies using various ketamine enantiomers and methods of administration, with particular attention to ketamine’s rapid and robust anti-suicidal effects in bipolar samples. This review determines that existing literature supports short-term ketamine use with bipolar depression, while cautioning that evidence is still limited for its adoption in recommended treatment algorithms.

Another promising area of ketamine research is addressed in “Anhedonia and depression severity measures during ketamine administration in treatment-resistant depression.” Researchers Kwaśny et al. analyzed antianhedonic response patterns in 28 TRD inpatients after short-term ketamine treatment, and found significant improvements in anhedonia in both responders and nonresponders. On this basis they argue for considering anhedonia as a symptom that can be effectively targeted for treatment with ketamine separately from depression.

In “Overcoming the myths of esketamine: Different and not difficult,” Buchmayer and Kasper, who are both experienced clinicians, review and evaluate literature on indication, preparation, administration, evidence-based management, and documentation of treatment with intranasal esketamine, including practical recommendations for an optimal clinical setting to reduce the risk of potential adverse effects. They argue that while this method of administration requires changes in conventional treatment procedures, these adaptations are uncomplicated and well worth the effort given evidence that esketamine as an add-on treatment results in beneficial therapeutic response in more than half of TRD patients, significantly improving their quality of life and overall functionality.

Whether ketamine is used as an add-on, a stand-alone therapy, or in combination with psychotherapy, its use can be enhanced with simple, cost-effective options—this is the case advanced by Purebl et al. in “Overcoming treatment gaps in the management of depression with non-pharmacological adjunctive strategies.” The authors point to the fact that drugs do not address all dimensions of a disorder, and supplemental strategies can fill some of these gaps. In addition to public education and workplace wellness programs to raise awareness, treatment can be augmented with in-person psychoeducation, online self-help tools, lifestyle counselling, and support from other patients and family members as well as from community facilitators such as religious figures, teachers, and non-clinical professionals. Non-pharmacological adjunctives have the advantage of potentially continuing beyond the end of treatment, thereby supporting the durability of therapeutic gains.

Since ketamine treatment is not yet standard care for many of the psychiatric disorders with which it has shown promise, systematic investigations are needed with diverse patient populations in preparation for wider use. In “Psychiatric and legal considerations for ketamine treatment within prison settings,” Bayrhammer-Savel et al. advocate for research focused on benefits and risks associated with ketamine treatment in prison populations. Their argument is that, given the high incidence of suicidality in these populations and the limited effectiveness of conventional antidepressants, such studies are warranted. Given the challenges of a prison context there is as yet limited evidence for its benefits and risks with this population; further research would serve as a step toward evidence-based use of ketamine with incarcerated patients.

Whatever the treatment context, it is assumed that a portion of response to any drug is placebo effect. In “Hype or hope? High placebo response in major depression treatment with ketamine and esketamine: A systematic review and meta-analysis,” Matsingos et al. report on their analysis of 14 randomized controlled studies of ketamine treatment in patients diagnosed with MDD. Their conclusion is that up to 72% of overall response to treatment can be attributed to placebo effect. While not discounting ketamine’s demonstrated robust antidepressant and anti-suicidal effects, this work points to the important role of a supportive context for any therapeutic application of ketamine.

Although the ketamine literature is already extensive, there are as yet few studies of its use in conjunction with psychotherapy, and evidence for safety and efficacy in applications beyond MDD is still modest (1). Numerous dimensions of ketamine’s impact and effects, alone or in combination with other interventions, remain to be examined. We hope these papers, in their diversity and curiosity about what lies beyond current knowledge, are harbingers of rich studies yet to come.

## References

[1] Hartelius G Muscat SA Bartova L. Editorial: Bridging the gap: an interdisciplinary perspective on ketamine in psychiatric disorders. Front Psychiatry. (2023) 14:1246891. doi: 10.3389/fpsyt.2023.1246891 37645640 PMC10461637

[2] Johnston J. N. Kadriu B. Kraus C. Henter I. D. Zarate C. A. Jr. Ketamine in neuropsychiatric disorders: an update. Neuropsychopharmacology. (2024) 49:23–40. doi: 10.1038/s41386-023-01632-1 37340091 PMC10700638

[3] Muscat S.-A. Hartelius G. Crouch C. R. Morin K. W. An integrative approach to ketamine therapy may enhance multiple dimensions of efficacy: improving therapeutic outcomes with treatment resistant depression. Front Psychiatry. (2021) 12:710338. doi: 10.3389/fpsyt.2021.710338 34899408 PMC8653702

